# Preparing to lead in polycrisis: a multi-method process evaluation of HR-driven psychosocial resilience training for leaders

**DOI:** 10.3389/fpsyg.2026.1900181

**Published:** 2026-10-23

**Authors:** Christina Hersom, André Torre

**Affiliations:** 1 Department of Educational Theory and Curriculum Studies, Educational Philosophy, and General Education, Danish School of Education, Aarhus University, Emdrup, Denmark; 2 VeldtofteStatistics, København, Denmark

**Keywords:** crisis leadership, emotional resilience, HRD, HRM, leader resilience, resilience training

## Abstract

In crises and fluctuating organizational contexts, resilience is increasingly recognized as a critical HRM strategy for sustaining organizational functioning and human capacity. This multi-method study evaluates an HRM-driven psychosocial resilience training program, including emotional training elements, for public and private leaders operating under polycrisis conditions. The study examines how leaders engage with resilience-oriented training and perceive its contribution to their capacity to anticipate, cope with, and learn from adversity. Participants´ evaluation of cyclic “resilience sprints,” the mechanisms of impact, and contextual influences were analyzed using descriptive and statistical methods. Findings show that structured resilience sprints support implementation, although the number, intervals, and duration of sprints were less suitable during polycrisis. Leaders achieved measurable effective training (Kirkpatrick reaction/learning levels), though training effectiveness (behavior/results levels) was more limited. Key facilitators included being gathered in a psychologically safe learning environment with committed trainers and supportive colleagues, while the main barrier was prioritizing training alongside work demands. Training-timing and motivation shifted dynamically throughout the period. The study contributes to HRM, Management, and Applied Resilience scholarship by highlighting overlooked dynamics and contextual factors shaping the build of leader resilience during crises, when most needed, and translates these into concrete considerations for HR practice.

## Introduction

Organizations and their leaders today face increasingly complex and interconnected crises (Boin and Lagadec, 2000; Riggio and Newstead, 2023; Uhl-Bien, 2021). Despite these challenges, organizations must still thrive through strategic human resource management (HRM) (Lengnick-Hall et al., 2011) or even gaining and grow from this disorder (Taleb, 2013). To adapt and function during constant system changes and unpredictable crises, organizations need to activate a human immune system that absorbs external stressors, learns from them, and strengthens the system moving forward, rather than relying solely on risk management and accidental solutions (Kupers and Kupers, 2014). Developing organizational resilience capacity to respond to current crises and adversity involves strategic HRM to create competencies among core employees that aggregate at the organizational level, enabling organizations to better respond to future crises (Lengnick-Hall et al., 2011; van der Vegt et al., 2015). More specifically, it requires capabilities to navigate significant uncertainties, paradoxes, and extreme conditions (Förster et al., 2022c; Giousmpasoglou, 2024; Giustiniano et al., 2020; Lewis and Smith, 2023; Lombardi et al., 2021; Ukhova, 2020; Wu et al., 2021). For leaders, such challenges can also provide “crucible moments,” offering important insights, higher adversity tolerance, and enhanced decision-making capabilities amid chaos (Breen, 2017). As resilience drives long-term performance and well-being, increased focus on this area is highly needed in management research (Hartmann et al., 2020; Lengnick-Hall et al., 2011). This study therefore positions resilience as a multifaceted managerial concept distinct from non-professional resilience, while acknowledging the need for integrating psychological resilience within leadership research (Daghmoumi and Zenjari, 2023; Mann et al., 2021). Resilience has predominantly been studied within psychological fields (Raetze et al., 2022) but identifying resilience facilitators and barriers in organizations is crucial for advancing Business, Management, and HRM knowledge to develop leader behavior under pressure during crises (Duchek, 2020; James et al., 2011). However, research on psychological resilience training for professional leaders is notably limited (Lombardi et al., 2021). A recent empirical review (*N* = 48) found no specific resilience training programs developed and evaluated for leaders, highlighting a critical gap in times of crisis (Scheuch et al., 2021). Given its strategic importance for organizational and individual outcomes in a context of constant change and adversity (Daghmoumi and Zenjari, 2023; Förster et al., 2022c), this gap is particularly noteworthy. Resilience development is paramount in organizations (Fletcher and Sarkar, 2013; Linnenluecke, 2017; McLarnon and Rothstein, 2013; Raetze et al., 2022; Stokes et al., 2019; Williams et al., 2017). Therefore, this study evaluates the design and implementation of an HRM-driven resilience training program for leaders. This paper explores how HR-business partners and HR-consultants strategically and operationally can support leaders in developing their cognitive, emotional, behavioural, and social resilience capabilities (Branicki et al., 2019; Richardson, 2002; Williams et al., 2017) during times of crises. This organizational supportive approach proactively shifts the responsibility for resilience towards strategic HRM practices (Daghmoumi and Zenjari, 2023; Duchek, 2020; Lengnick-Hall et al., 2011). By evaluating resilience training processes at managerial levels, this study deepens the understanding of building leader resilience capabilities, enabling leaders to adapt to complex pressures and effectively lead themselves and others, rather than denying or retreating from adversity. Today leaders need to face a world of geopolitical wars, environmental disasters, cybersecurity threads, or more indirect mouldering disorders turning little by little into a crisis. Positioned within crisis leadership, psychological resilience, and leadership development research (Giustiniano et al., 2020; Lombardi et al., 2021; Uhl-Bien, 2021; Williams et al., 2017), this integrative study offers empirical insights into the nature of organizational leader resilience training during polycrisis circumstances. A polycrisis is defined here as simultaneous, complex crises that are both entangled and interacting, with no single causes and no simple fixes, making it difficult to grasp the scale of the transformations unfolding. Because the whole is more overwhelming than the sum of its parts, it threatens the subject’s ability to cope and, therefore, the subject’s identity (Tooze, 2022). For example, the private-sector leaders in this study experienced COVID-19 restrictions, market-demand disruption, and supply-chain volatility not as separate pressures but as interacting ones: pandemic restrictions constrained production capacity at the same time as demand and supply patterns were shifting unpredictably, so that decisions made to manage one pressure (e.g., staffing under restrictions) directly limited leaders’ ability to respond to another (e.g., meeting fluctuating demand). This illustrates how, in a polycrisis, crises compound and interact across priorities and decisions rather than occurring in isolation. The process evaluation focuses on design, implementation, and contextual factors affecting leaders, thus unfolding multilevel organizational perspectives as requested (Raetze et al., 2022; Scheuch et al., 2021). Context may shape the enactment of resilience development (Linnenluecke, 2017) and is therefore vital to evaluate in complex human interventions, holding the potential to explain variations in health and performance outcomes (Moore et al., 2015).

### Defining leader resilience

Resilience can be seen both as a human capacity and as an observable outcome when dealing with adversity (Britt et al., 2016). From an organizational perspective, resilience has been conceptualized as a meta-capability involving anticipating, coping, and adapting responses in adversity (Duchek, 2020). The current study builds on these views by focusing on training and development processes that potentially cultivate the capacity for resilience, rather than solely on the demonstration of health and performance outcomes (Britt et al., 2016). This mirrors large-scale institutional resilience programs such as the U.S. military’s Comprehensive Soldier Fitness program, which similarly aimed to shift focus from trauma and disorder toward preventive, ordinary resilience and personal growth, and was proposed as a model transferable to other large and challenged institutions (Cornum et al., 2011). By integrating crisis management and resilience research, resilience in this paper refers to the process by which leaders build and use their capability endowments to interact with the environment in a way that positively adjusts and maintains functioning prior to, during, and following adversity (Williams et al., 2017). This adapted process definition of resilience to organizational environments incorporates the notion of antifragility (Taleb, 2013). It is operationalized as “intelligent training of leader resilience”, focusing on iteratively developing and applying flexible human strategies to lead in rapidly changing and uncertain environments by addressing adversities and learning from them. Participants must train collectively and individually to gain flexibility from diverse, adverse-based micro-processes at work, to better deal with futures challenges. Methodologically, experiential learning (Kolb, 2014), in this study, aims to train various resilience behavior, emphasizing the interaction between individual leader characteristics and situational workplace factors focusing on self-awareness, self-reflection and appropriate coping strategies (Förster et al., 2022b). In this study, psychosocial resilience follows Vanhove et al., 2016 usage across training studies: resilience-building programmes typically target the psychosocial subset of protective factors, which is commonly self-efficacy, optimism, social resources, and cognitive appraisal/coping. Consistent with this, and with psychosocial curriculum-design approaches that select context-relevant factors from a broader evidence base (Ungar and Jefferies, 2021), the organizations in this study composed their final sprint curricula by selecting resilience-promoting content suited to their respective adversity contexts, targeting the behavioral (strength-based), cognitive/emotional (mental-based), and social (relational-based) dimensions of psychosocial resilience introduced above.

## Materials and methods

### Program theory

While intervention mechanisms can be contextually appropriate for inducing change, their outcomes often vary due to external factors (Moore et al., 2015). These factors, both pre-existing and emergent, can either hinder or support the intervention effectiveness. Here, the methodology enables a specific analysis of these non-causal effects, offering new insights into resilience training’s less understood aspects (Moore et al., 2015; Raetze et al., 2022). Figure 1 illustrates the study’s´ program theory, serving as the detailed roadmap of the intervention and its components, where the process evaluation is specifically focusing on key components highlighted in green.

**Figure 1:**
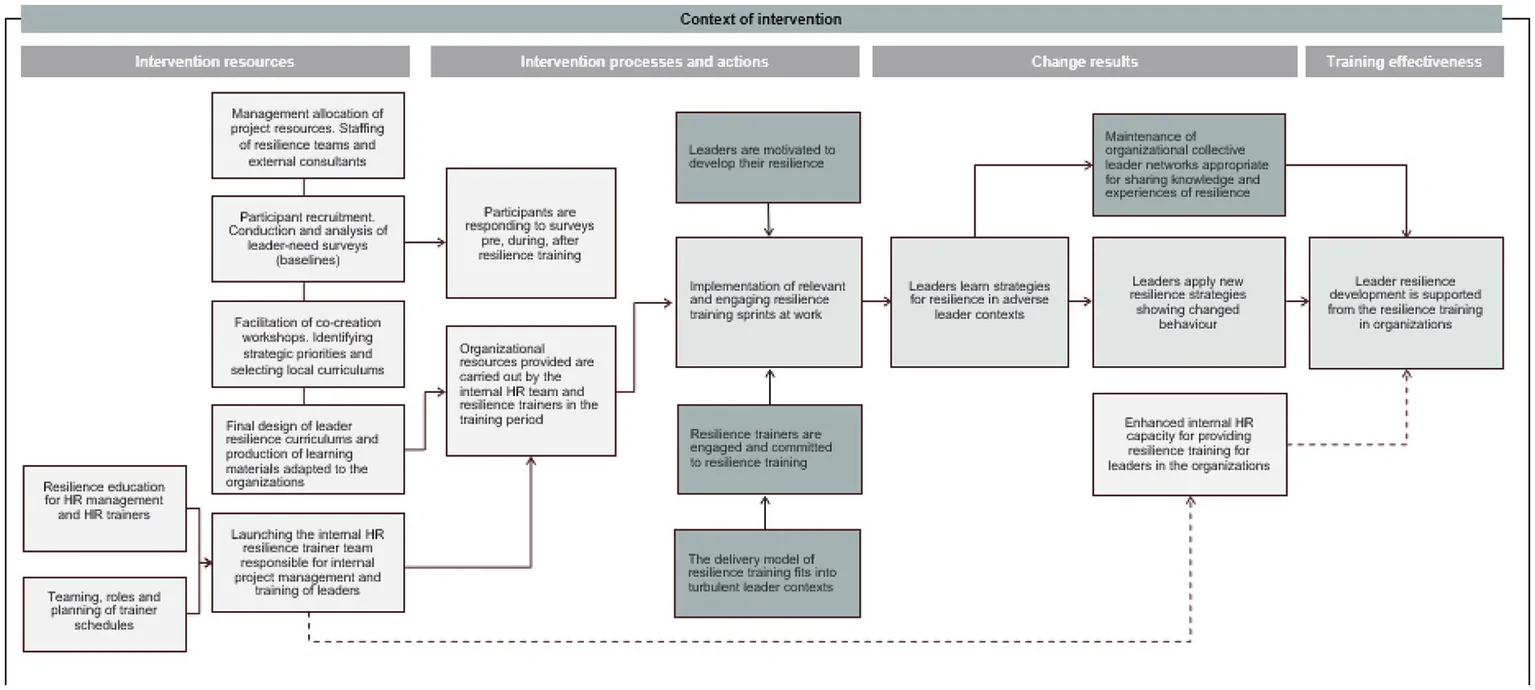
Program theory for leader resilience training in organizations.

### Theoretical framework for the process evaluation

The study uses the Medical Research Council (MRC) framework, emphasizing the context’s role in complex human interventions to interpret and generalize findings (Moore et al., 2015). It accounts for nonlinearity and potential reversals due to positive or negative feedback loops during implementation (Pawson et al., 2014) and identifies mechanisms linking intervention processes to outcomes and variations (Moore et al., 2015; Nielsen and Abildgaard, 2013; Randall et al., 2007). Figure 2 translates the subset of program theory components highlighted in green in Figure 1, which is those selected for process evaluation, into the three assessable components evaluated in this study: implementation, mechanisms of impact, and the context of the leader resilience training.

**Figure 2:**
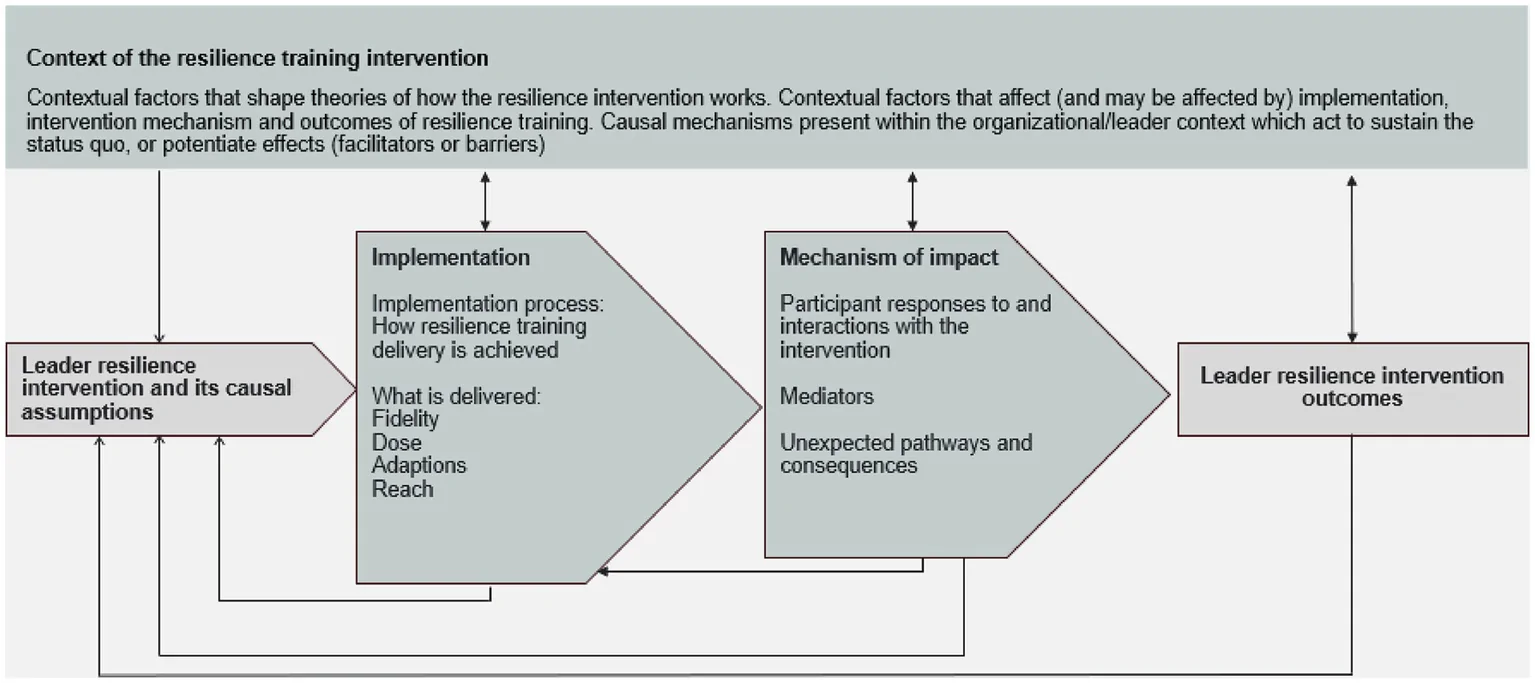
Process evaluation framework, adapted from Moore et al. (2015), under CC BY 4.0. Visual design and colours modified and findings from the present study added.

The process evaluation framework was tailored to explore the interrelations among resilience training components and key functions. Yet, it remained flexible to incorporate emerging inquiries as per established guidelines (Moore et al., 2015). Concretely, the three process evaluation components in Figure 2 (implementation, mechanisms of impact, and context) are not assessed in isolation but are treated as interdependent. For instance, implementation-fidelity and contextual facilitators/barriers are expected to shape how mechanisms of impact unfold and are interpreted. Given the longitudinal design, the reverse pathway, in which leaders’ ongoing evaluations of training mechanisms plausibly reinforce or erode contextual factors such as motivation throughout the program, is also plausible, though not directly tested here. Each component is nonetheless assessed with an analytic technique matched to its specific evaluative purpose. This means that descriptive statistics characterize implementation fidelity and contextual facilitators and barriers. The mechanisms of impact component combines descriptive analysis of Kirkpatrick evaluations with a chi-square test of between-organization variation and a Markov chain model examining whether short-term evaluation patterns are consistent with longer-run trajectories, under the simplifying assumptions of stationarity and the first-order Markov property.

### Study design

The evaluation included two major Danish organizations, both of which are nationally and internationally recognized for their constant progress and significant contributions. By examining prolonged psychosocial resilience training for leaders across these pressured organizations during extreme times, this pioneering study answers calls for prospective longitudinal designs in organizational resilience training (Britt et al., 2016; Vanhove et al., 2016) at managerial levels (Scheuch et al., 2021). It explores resilience as flexible self-regulation (Bonanno, 2021) in dynamic processes (Fletcher and Sarkar, 2013; Masten, 2001), vital in stressed and crisis-impacted leader environments (Duchek, 2020; Förster and Duchek, 2022; Ledesma, 2014; Ukhova, 2020). The multi-method process evaluation focuses on the implemented program, learning processes and self-reported development experiences over time.

### Setting and sample

The core training program coincided with severe polycrisis conditions, blurring the boundaries between crises in time and space. The public-sector organization operates in acute, round-the-clock human-facing service provision, whose leaders faced concurrent COVID-19 pressures, sector-wide labor actions, and mandatory compensatory time-off obligations during the study period; while necessary given widespread staff exhaustion, this compensatory leave simultaneously reduced available staffing and further increased pressure on the leaders who remained on duty. The private-sector organization operates in a production and supply-chain-dependent industry producing a critical, globally-demanded product. During the study period, leaders faced acute demand rushes driven by international build up stock’s behavior, disrupted global freight and logistics capacity, periodic shortages of essential production inputs, and the operational challenge of maintaining continuous production through strict internal infection-control measures (e.g., isolated shift teams to contain outbreaks without stopping output). Both organizations retained full leadership complements throughout, but operated under sustained, compounding external adversity rather than a single discrete crisis event. This constellation of concurrent crisis pressures constituted the study’s research context. Lockdown crises conditions yielded 48 participants for this study: 20 public-sector leaders and 28 private-sector leaders from two Denmark-based organizations who completed surveys and evaluated resilience sprints during training. Internal HR-resilience trainer teams were pre-educated based on the final locally designed resilience training curriculums as part of a highly involving research approach. Studies (Baker et al., 2021; Lester et al., 2011) demonstrate that trainers who utilize resilience skills can effectively foster trust and safety among participants, enhancing their engagement and motivation for lasting behavioral changes.

### The leader resilience program

The study’s methodology integrated crisis and resilience streams to adapt effectively to reality (Williams et al., 2017). The training program proactively aimed to provide insights and response opportunities for leaders while facing ongoing adversities in the extreme pressured working environments. First, the research was based on integrative meta-theories of resilience in organizational environments, emphasizing process-oriented and developmental approaches to resilience-building (Britt et al., 2016; Cooper et al., 2013). Second, the resilience training program design was informed by criteria identified through systematic reviews of psychological resilience-building programs (Ijntema et al., 2019), meta-analytic reviews of resilience-building programs in organizational settings (Robertson et al., 2015; Vanhove et al., 2016), and precedents such as the Comprehensive Soldier Fitness program addressing pressured environments (Lester et al., 2011). The reviews highlighted resilience training’s potential effectiveness but also noted the need for more research. Especially, the lack of research on psychosocial leader resilience training was evident. Third, resilience factors identified in leader contexts (Förster and Duchek, 2017; Ukhova, 2020) were reviewed. Finally, through adapted experiential learning methods (Kolb and Kolb, 1984), the study offered multimodal psychosocial leader resilience training, adopting a wide range of adversities the participants faced as leaders. The leader resilience program targeted cognitive, emotional, behavioral, and social resilience strategies (Richardson, 2002; Williams et al., 2017) as various mechanisms for leading oneself and others during the crisis-laden conditions.

As recommended for local involvement and flexible resilience curriculums (Ungar and Jefferies, 2021), the two organizations engaged in 20 composed resilience sprints. Both organizations completed six sprints of strength-based resilience training. Cognitive resilience training included six sprints in the public-sector organization and ten in the private sector, while emotional/relational resilience training included eight in the public-sector and four in the private-sector organization.

### Multi-method process evaluation approach (procedure)

A process-focused approach is crucial in resilience interventions, particularly for training and education in organizations (Smaliukiene et al., 2024). This study evaluates the implementation of hypothesized change mechanisms in processes during a longer period of resilience training (the what) and how it works for leaders (for whom) influenced by contextual factors (under which circumstances). Given the sensitivity of human interventions to implementation and contextual influences, we used method triangulation by including three different datasets in the study to enhance reliability of the study. This multi-method approach and the collection of both formative and summative data, strengthen validity of findings by synthesizing information from various data sources (Grant et al., 2013; Miles and Huberman, 1994). For practical use, an adapted multi-method matrix (Farmer et al., 2006; Miles and Huberman, 1994) was used to systematically overview the process including data sources, their uses, evaluation types, data collection methods, sample details, analysis techniques, and the framework components they cover. This matrix, shown in Appendix 1, aligned and integrated the Program Theory (Figure 1) with the process evaluation framework (Figure 2).

### Measurements and analysis strategies

To cover the three components of the evaluation framework, different survey instruments were applied in the study, as illustrated in Appendix 2. Consistent with the process evaluation aim of this study, the analytic approach is predominantly descriptive, supplemented by inferential tests (chi-square) and exploratory modelling (Markov chains) only where these serve to characterize implementation and mechanism patterns rather than to test the intervention effectiveness.

#### Component 1: the resilience training implementation

Reviewing resilience training, it is challenging to pinpoint the most effective format (Robertson et al., 2015) as programs vary greatly in content, delivery, and length (Joyce et al., 2018). Therefore, the research team assessed the appropriateness of the long-term resilience training format, applied to grab continuous adverse and pressured leader contexts. Following recommendations to develop tailored questionnaires for evaluating unique aims and objectives in complex interventions (Arends et al., 2014; Moore et al., 2015), we created a brief 4-item questionnaire. The four items targeted the structural parameters of the sprint format specifically (number of sprints, interval between sprints, duration per sprint, and distribution of within-sprint elements), rather than resilience content or outcomes. Descriptive statistical analysis was conducted using SAS statistical software for evaluating this. Internal trainers documented participant attendance, while the main researcher monitored protocol compliance and curriculum fidelity. The researcher additionally logged larger implementation adaptations required due to crisis restrictions or training conditions for review and discussion.

#### Component 2: the resilience training mechanism of impact

Through a realistic approach, mechanisms can be seen as elements of how participants reason and react to available resources in the context, facilitating changes within an open system of space, time, and social relationships (Lacouture et al., 2015; Pawson et al., 2014). Here, the provided resilience resources relied on leaders’ responses to facilitated sessions and self-directed interactions in adverse work situations. The research team assessed these resilience training mechanisms through “leader resilience sprints” designed as weekly experiential learning cycles based on experiential learning theory (Kolb and Kolb, 1984).

Participants evaluated the training weekly at the end of each facilitated and self-directed resilience sprint, with data collected through posters or mobile surveys depending on the different crisis restrictions. To minimize participant burden in process data collection (Moore et al., 2015), we applied a tailored and practical version of the Kirkpatrick Model (Alsalamah and Callinan, 2022; Kirkpatrick, 2022) suited to intensive training sessions. The model covered two dimensions and four levels: Effective training, including (1) reaction—leaders` immediate reactions and satisfaction, and (2) learning—gains in valuable knowledge and insights; and Training effectiveness, including (3) behavior—application of new practices, and (4) results—resilience development attributable to supportive training. As training occurred concurrently with live polycrisis conditions, sprints drew on both adversity leaders had directly experienced during the preceding week and anticipated (‘what if’) future scenarios to move away from linear thinking and better understand external change (Van der Heijden and Heijden, 2005). Level 4 (results) therefore reflects resilience development attributed by participants to a combination of demonstrated behavior in ongoing adversity and rehearsed responses to anticipated future adversity, rather than a purely prospective, untested capacity (Kuntz et al., 2017). We note that Level 4 here refers to outcomes tied to the training’s own stated goal, which is leader resilience development, rather than organizational or business performance results, consistent with Kirkpatrick’s original framework. As with Levels 1–2, this item relies on leaders’ self-report and is not independently verified by observed behavior or a validated resilience measure; it should therefore be weighted accordingly when interpreting the strength of the Level 4 findings (see Limitations). An open-ended response option was added for additional comments. The evaluation validity of the Kirkpatrick model has been demonstrated in a comprehensive meta-analysis (Lacerenza et al., 2017) including 335 leadership training studies (*N* = 26.573), showing significant variance in training effectiveness based on design, delivery, and implementation. SAS statistical software was used for descriptive analysis of the distribution of Kirkpatrick levels during the training period. A chi-square test, an inferential statistical test, was indicating the distribution across the three overall implemented resilience factors to expand the understanding of its relevance.

Further, to explore the dynamic and optimal length of future leader resilience programs, we used a Markov chain model (Spedicato, 2017) to demonstrate evaluations of training mechanism over time. In this formulation, the four Kirkpatrick levels were treated as sequential and interconnected states in a dynamic process, where participants transition sequentially from one level to the next (Kirkpatrick 1–4). By the Markov property, the future state depends only on the current state, not on the path taken. Drawing this analogy allowed us to estimate transition probabilities between evaluation levels and, in turn, to predict participants` progression as change mechanisms if the training were extended, based on the data feasibly collected within the study period. To our knowledge, this is a pioneering statistical method in both resilience and leadership intervention research using R Version 4.2.3 (R Core Team, 2024) and the Markov chain package (Spedicato, 2017). This Markov chain approach is a predictive or projective technique, estimating long-run transition probabilities based on observed short-term evaluation patterns under the simplifying assumptions of stationarity and the first-order Markov property. Its value here lies in generating concrete, testable projections of how evaluation patterns might unfold over a longer training period—projections that can directly inform the design and duration of future longitudinal resilience studies. These assumptions could not be formally tested with the available data, given the reduced number of transitions following exclusion of missing responses (see Results). The resulting projections should therefore be read as indicative rather than as robust forecasts of intervention effectiveness (see also Limitations). Considering other change mechanism, the main researcher additionally logged notable participant engagement, unexpected pathways and consequences for review and discussion of the intervention.

#### Component 3: the contextual factors

Facilitators or barriers present in individual, collective, or organizational contexts may influence implementing research in practice and intervention mechanisms (Rycroft-Malone et al., 2011). Here, the research team evaluated how context influenced resilience training processes and leaders’ self-reported resilience development. By integrating contextual factors from the program theory with themes emerging from initial training, it allowed the development of relevant evaluation questionnaires, which is crucial for process evaluation of complex interventions (Moore et al., 2015). The questionnaire comprised eight discussed items. A repeated measurement was then conducted during (Time 2) and post-training (Time 3) to examine contextual factors and capture changes over time in the leader context. Using SAS statistical software, we conducted a descriptive analysis of contextual barriers and facilitators as reported by participants. Additionally, the main researcher logged notable contextual research and project management issues for review and discussion.

In sum, the multi-method approach enabled a multifaceted three-part evaluation of the resilience intervention, enhancing the understanding of implementation processes, mechanisms of impact, and contextual influences on leaders’ self-reported resilience development in real-world crisis settings marked by compounding adversities and stressful pressure.

### Data triangulation strategy

To keep an overview of the 3 component results, we applied a convergence coding matrix (Farmer et al., 2006) to compare findings across data sources, categorizing relationships as agreement, partial agreement, silence, or dissonance. Combined with notable observations during the period, this enables a nuanced discussion of complex interventions (Moore et al., 2015) and highlights the dynamic nature of systemic and human resilience pathways (Masten and Reed, 2002; Sarkar and Fletcher, 2014).

### Methodical soundness

We aimed to ensure trustworthiness in the study (Krefting, 1991). Transferability (external validity) was strengthened by adapting the curriculum backlog to local baseline data and internal HRM knowledge. Dependability (reliability) was supported through the education of internal resilience trainer teams, standardization of training methods, and coordinating with external consultants when needed. Credibility (internal validity) was enhanced by developing contextual questionnaires based on early observations and discussions, enabling the capture of emergent factors during extreme conditions and allowing tests across organizations. Confirmability (objectivity) was ensured by using diverse data sources, storing blinded survey data in Survey X-act for independent statistical analysis, and applying recognized frameworks to minimize biases.

### Ethical considerations

Approval of the intervention was obtained from HR management and department managers in both organizations. Participation was voluntary and confidential, and leaders were informed of their right to withdraw at any time. Informed consent was obtained from each participant via Survey X-act prior to completing any questionnaires.

## Results

Participant numbers for the intervention group surveys declined as expected under the turbulent organizational conditions: from 48 leaders at pre-intervention, to 41 during the intervention, and 36 at post-intervention (< 3 months). This corresponds to sequential drops of 14.6 and 12.2%, and an overall attrition rate of 25.0%. Participant characteristics across the three time points are presented in Table 1.

**Table 1:** Participant characteristics across survey time points.

Variable	Categories	Time 0	Time 1	Time 2
Organization	Private	28	23	22
	Public	20	18	14
Gender	Female	30	25	22
	Male	18	16	14
Age		13	10	9
	40–50	19	17	16
	> 50	16	14	11
Education	Long	33	28	25
	Medium	15	13	11
Responsibility	Yes	25	20	16
	No	23	21	20
Total		48	41	36

Counts reflect the number of participants responding at each time point, while differences in N across time points are due to attrition.

### Component 1: implementation

In total, 35 leaders participated in this post-evaluation phase of the study, yielding a response rate of 97%. Across items, a substantial proportion of leaders (20–37%) selected the neutral midpoint (“either disagree/agree”). Specifically, 20% found the number of sprints appropriate, while 31% considered both the interval between sprints and the duration of each sprint suitable. Nearly half of the participants (49%) evaluated the distribution of sprint elements within each session as appropriate. These results are presented in Table 2.

**Table 2:** Descriptive statistics for participants’ evaluations of the resilience sprint design (*N* = 35).

Statement	Disagree % (n)	Either disagree/agree % (n)	Agree % (n)	Mean	SD	Median
The number of sprints in the resilience course (20 sprints) has seemed appropriate to me	43	37	20	1.77	0.74	2
The interval between sprints (approx. 1 week) in the resilience course has been appropriate for me	43	26	31	1.88	0.86	2
The duration of a sprint (60 min) has been appropriate for me to spend on resilience training	49	20	31	1.82	0.86	2
The distribution of the sprint elements (joint review of the past week, new knowledge, time with my training book and dialogue with others) has been appropriately distributed over the 60 min	17	34	49	2.32	0.76	2

Response categories were coded as 1 = Disagree, 2 = Either disagree/agree, and 3 = Agree. The response categories combined “Strongly Disagree” with “Disagree” and “Strongly Agree” with “Agree” for analysis.

Taken together, these ratings suggest that the overall sprint structure was perceived as the strongest component of the implementation, whereas the number, interval, and duration of sprint sessions were viewed as, comparatively, less appropriate in relation to participants’ preferences.

### Component 2: impact of mechanism

Using Kirkpatrick’s evaluation framework, this section begins with a descriptive analysis of the ongoing training evaluations, then including the resilience factors targeted by the program. We then present results from a Markov chain model that predicts the outcomes if the training program were extended. In total, 42 leaders completed 20 “resilience sprints” across the two organizations, with 18 participants from one organization and 24 from the other.

First, the total descriptive analysis of the weekly Kirkpatrick evaluations showed that leaders reported effective training (Levels 1–2) to a substantially greater extent (78%) than training effectiveness (Levels 3–4) (21%). In the public-sector organization, evaluations were distributed as 31% reaction, 54% learning, 11% behavior, and 4% results. In the private-sector organization, the distribution was 46% reaction, 29% learning, 11% behavior, and 15% results, indicating that the two organizations emphasized different aspects of the training process and resilience sprints.

Second, to examine how these evaluations aligned with the implemented resilience factor categories, a chi-square test was conducted. For strength resilience, no significant difference was found (*p* = 0.3169), suggesting relatively consistent evaluation patterns across organizations. For mental resilience, a significant difference emerged (*p* < 0.0001), indicating divergent evaluation patterns between organizations. For relational resilience, the distribution approached significance (*p* = 0.0582), suggesting some variation but not at a conventional significant threshold. Table 3 provides an integrated overview of these distributions.

**Table 3:** Kirkpatrick evaluation results by organization and category.

Organization	Category	Reaction	Learning	Behavior	Results	Total
Public	Strength	33%	49%	12%	6%	51
	Mental	31%	62%	4%	2%	45
	Relational	30%	50%	18%	2%	44
	Total	31%	54%	11%	4%	140
Private	Strength	29%	43%	11%	17%	63
	Mental	54%	20%	11%	14%	98
	Relational	57%	24%	10%	10%	21
	Total	46%	29%	11%	15%	182
Overall	Strength	31%	46%	11%	12%	114
	Mental	47%	34%	9%	10%	143
	Relational	38%	42%	15%	5%	65
	Total	39%	39%	11%	10%	322

The distribution of weekly Kirkpatrick evaluations (Levels 1–4) across the two organizations and the alignment analysis assessed with a chi-square test.

Finally, to examine change mechanisms and predict potential outcomes in an extended program, the Markov chain model was applied. Due to a 38% response rate, transitions involving missing data were excluded to strengthen validity. For strength resilience, long-term predictions suggest that approximately 32% of participants would remain at Level 1 (reaction), 41% at Level 2 (learning), 13% at Level 3 (behavior), and 14% at Level 4 (results). For mental resilience, long-term predictions indicate 52% at Level 1, 32% at Level 2, 7% at Level 3, and 9% at Level 4. For relational resilience, the transition matrix formed what would conventionally be interpreted as an absorbing Markov chain, meaning that participants who reached Level 4 (results) appeared to remain there. However, no transitions were observed departing from Level 4 in this category (*n* = 0). Because the Markov chain package assigns a self-transition probability of 1 to states with no outgoing data, this “absorbing” pattern is a modeling artifact of missing data, not evidence that participants remained at Level 4. This finding should not be interpreted as a genuinely absorbing state and warrants replication with a larger sample before further weight is placed on it.

### Component 3: contextual factors

In total, 35 leaders participated in the repeated evaluation part of the study, yielding response rates of 85% during the intervention and 97% post-intervention, enabling a valid examination of contextual factors over time. Table 4 presents survey response distributions, including percentages of participants who disagreed, were neutral, or agreed with statements about the training’s effectiveness, timing, prioritization, colleague acceptance, atmosphere, motivation, and trainer engagement:

**Table 4:** Response distributions for contextual factors at T2 and T3.

Statement	Time	Disagree %	Neutral %	Agree %
It has worked well for me that we have been gathered for the resilience training	T2	14	23	63
	T3	14	23	63
The time of day we have met has suited me well	T2	31	17	51
	T3	23	43	34
I have been able to prioritize resilience training without significantly disrupting my work	T2	46	23	31
	T3	57	11	31
I have experienced acceptance from my colleagues when I have had to leave for resilience sprints	T2	6	34	60
	T3	0	43	57
I have experienced a free and open atmosphere when we have shared knowledge and experiences about resilience	T2	6	20	74
	T3	6	17	77
I have been motivated to develop my resilience during the course	T2	11	29	60
	T3	14	46	40
The external trainer has seemed engaged in the training	T2	7	7	85
	T3	—	—	—
The internal trainers have been committed to the training	T2	9	31	60
	T3	6	17	77

The response categories combined “Strongly Disagree” with “Disagree” and “Strongly Agree” with “Agree” for analysis.

Particularly, agreement was high for statements about being gathered as leaders for resilience training, working well, experiencing acceptance from colleagues, and a free atmosphere for sharing resilience knowledge and experiences. However, fewer participants agreed upon the timing of training (decreased further overtime) and only one third agreed they could prioritize training without disrupting work. Motivation to develop resilience was initially strong (60%) but dropped substantially over time (40%). The engagement of external trainers who were only supporting the initial onboarding of internal trainers was reported (at T2), with very high agreement, while internal trainers showed increased high commitment at T3. Detailed response transitions for all contextual factors, illustrating both distributions and changes over time, are presented in Appendix 3.

### The total process evaluation

In total, within the 3-component evaluation, shown in Figure 3, all findings from the statistical analysis are organized within the study’s applied framework:

**Figure 3:**
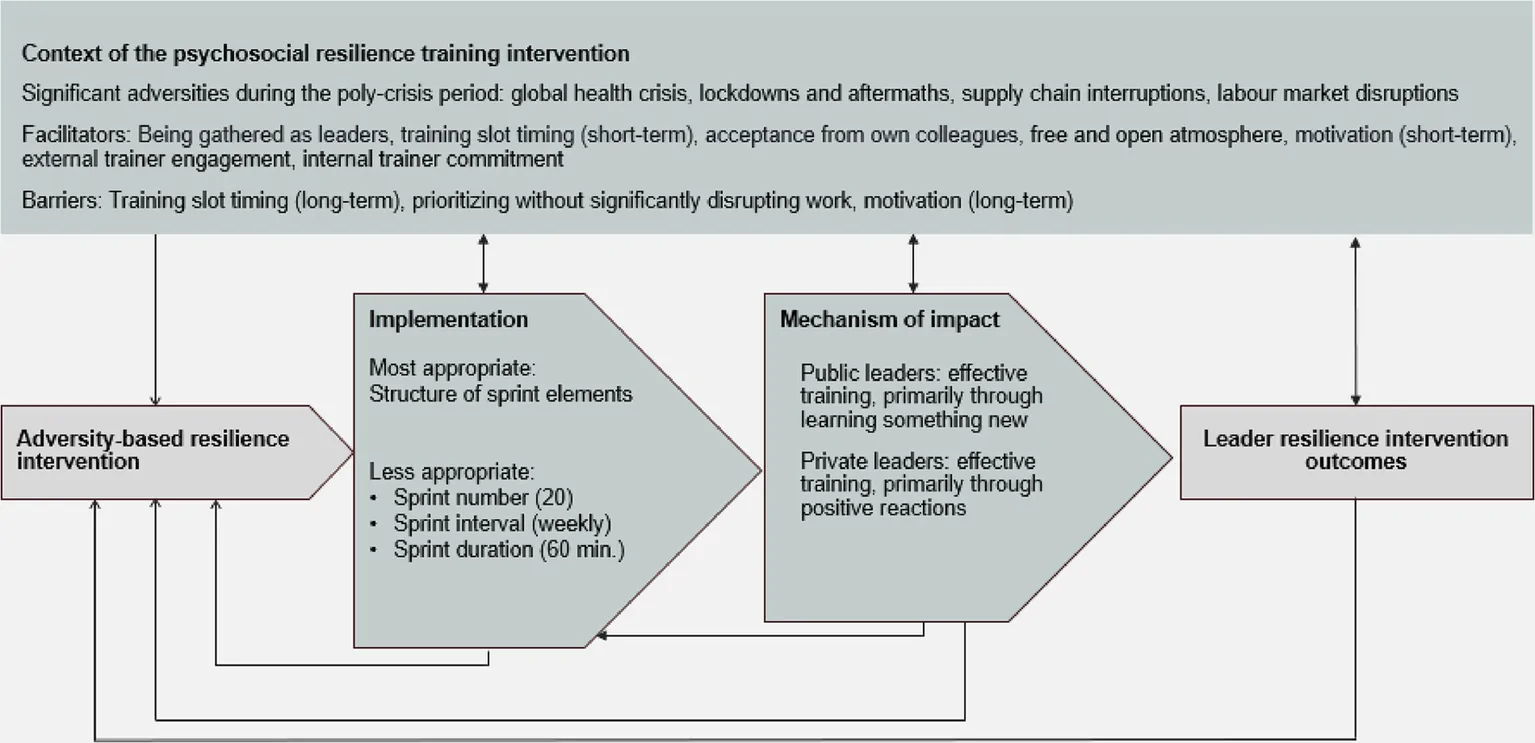
Results of the leader resilience training intervention, presented within the process evaluation framework adapted from Moore et al. (2015), under CC BY 4.0. Visual design and colours modified and findings from the present study added.

Findings indicated that the pre-designed sprint structure functioned as the primary facilitator of implementation, whereas the number, intervals, and duration of sprints became influential barriers in a long-term training program. Leaders predominantly experienced effective training through positive session reactions and learning something new during the weekly resilience sprints, with mental training being the most variable component and strength-based sprints appearing more supportive of participants’ self-reported resilience development. Key contextual facilitators included trainer commitment and being gathered in a supportive, psychologically safe training environment, while the main contextual barrier was the difficulty of prioritizing resilience training without significantly disrupting work. Over time, the training schedule shifted from being perceived fit to becoming a barrier. The most notable change observed was a decline in motivation during the pressured period of resilience training.

## Discussion

The results from this process evaluation are discussed within the study’s applied framework of implementation, mechanisms of impact, and contextual influencers, to illuminate how the program was implemented and experienced within crisis-affected organizational settings.

### Implementation

The pre-designed sprint structure and organized sessions generally facilitated implementation, whereas the number, intervals, and duration of sprints were more difficult to integrate into crisis-affected work contexts. This aligns with calls for examining longitudinal resilience training (Britt et al., 2016; Scheuch et al., 2021), as resilience develops over time through repeated engagement with adversity (Masten and Wright, 2010). In this study, the training process unfolded, both squeezed and extended, over nearly six months, adapting to ongoing global and organizational crises and the imposition of restrictions, which placed additional demands on participants.

Post-evaluation results indicated that the 20-sprints format functioned as a barrier for some leaders, although many responded neutrally. Prior research shows considerable variation in intervention length (Scheuch et al., 2021; Vanhove et al., 2016), raising the question of how long leader resilience development activities should last when implemented as a strategic HRM activity (Bardoel et al., 2014; Branicki et al., 2019). While fewer sprints may be more feasible in pressured leader environments, reducing the duration could limit opportunities for collective and experiential learning emerging directly from ongoing crises.

Although the interval and brief duration of sessions were also noted as barriers, as substantial proportion of participants remained neutral. Repeated application of learned skills is central to effective resilience training (Scheuch et al., 2021), suggesting that alternative learning cycles may warrant exploration. The sprint elements—discussions of experienced resilience strategies, self-evaluation, theoretical input, reflection, and anticipation for the next sprint—occurred as key facilitators, enabling leaders to anticipate, cope with, and learn from real-life adversity in line with experiential learning theory relevant to resilience development (Breen, 2017; Förster and Duchek, 2022; Kolb, 2014). Experiential learning theory frames learning as a core adaptive process in which the leaders engage dynamically with their environment and convert lived experiences into usable knowledge.

The flexible, organization-wide, multimodal design supported individual learning needs (Cheshire et al., 2017), yet this flexibility (multimodal options, rescheduling, and redesign) also increased workload for stakeholders. Unexpected crisis-driven shifts, such as sudden transitions to virtual delivery, logistical disruptions including unexpectedly transport to off-site locations, or obligatory time off in lieu due to intensive work during the period, required additional resources and highlighted the need for agility among designers, implementers, and participants. These conditions mirror the polycrisis context in which leaders operate, reinforcing that long-time resilience training itself must embody structural and supportive resilience.

Overall, the results suggest that a perfect-fit resilience design may be unrealistic in high-demand, continuously changing leader contexts. Instead, resilience interventions must function as organizational, social, and human adaptive systems capable of reorganizing in rapid response to emerging challenges, just as leaders are expected to do. The program’s implementation thus reflects the very conditions it seeks to address, transforming complex and fluctuating realities into opportunities for learning, development, and enhanced thriving and performance.

### Mechanisms of impact

Using Kirkpatrick’s evaluation framework, this section discusses the integration of ongoing training evaluations, the resilience factors targeted by the program, and probabilistic outcomes if the training program were extended. In total, 42 leaders completed 20 “resilience sprints” across the two organizations, with 18 participants from one organization and 24 from the other.

First, focusing on leaders’ interactions and responses to the resilience sprints, the findings show that participants reported effective training (reaction and learning) to a markedly greater extent than training effectiveness (behavior and results). Public-sector leaders primarily reported learning something new from the sprints, whereas private-sector leaders more often expressed positive reactions yet showed the highest proportion of resilience development supported by the training. This suggests that leaders engage with and benefit from resilience training in different ways, depending on their organizational context and the types of adversity they face.

Interestingly, several participants struggled to recognize or self-evaluate their resilience development, even when they could articulate newly applied strategies in peer conversations. This indicates that high-quality reflection and explicit linking of resilience concepts, processes, and weekly outcomes are critical for both resilience learning and evaluation. Not all experiences and actions inherently lead to learning (Schoen, 1992; Winter and Burroughs, 1989), which may help explain why resilience, which is still a relatively new HR concept in leadership development, prompted confusion and neutral self-evaluations among some leaders. For instance, one public-sector leader expressed this uncertainty when hesitating in front of an evaluation poster: “But… how do I dare to score it? How do I know if I have developed my resilience?”. This illustrates not only the complexity of resilience as a construct, but also the specific challenge of assessing any resilience development because of training at Kirkpatrick Level 4. This difficulty may also be part of why leaders’ evaluations concentrated more heavily at Levels 1–2 (78%) than at Levels 3–4 (21%), as reaction and learning can be reported relatively directly, whereas judging one’s own resilience development asks for a more reflective form of self-assessment, one that, as the example above suggests, may not come easily to all participants. This raises the possibility that the observed Level 3–4 distribution reflects, at least in part, the difficulty of understanding and self-assessing psychosocial resilience, and not only the absence of behavior changes or development itself. Because transforming experiences into learning and demonstrable resilience requires reflective, second-order processes (Argyris et al., 1978; Schoen, 1992), the example also underscores the difficulty of distinguishing potential from manifested resilience in real-world training contexts (Johansson et al., 2018). Further, this may illustrate the broader challenge of separating resilience capacities and processes from their realized outcomes in health and performance (Britt et al., 2016). Beyond purposes and learning goals, clarifying what constitutes operational psychosocial resilience processes and potential outcomes is therefore essential for leaders engaged in experiential training during crisis affected times. Evaluations must be understandable, meaningful, and sensitive to emerging changes in mental, emotional, and behavioral responses to leading oneself and others through adversity. How this can be captured and measured consistently in a brief, practical way during training should be tested further in future resilience studies.

Second, the triangulated statistical analysis revealed a significant difference in mental resilience evaluations between organizations. This may reflect differences in cognitive appraisal, one of the psychosocial protective factors underpinning resilience in this study’s own framework (Vanhove et al., 2016), rather than differences in underlying resilience capacity. Public-sector leaders confronted acute, human-facing crises with continuously shifting, unpredictable operational demands and limited managerial support, consistent with evidence that such conditions increase decision complexity and personal strain for public leaders during the crisis period (Hølge-Hazelton et al., 2021), and with broader findings of the intensifying demands facing leaders’ resilience during crises (Förster et al., 2022a). These conditions have likely made it more difficult to cognitively process and apply resilience strategies during continuous weekly sprints. Private-sector leaders, by contrast, faced acute pressure to scale up delivery volumes rapidly amid sudden crisis demand surges, but this operational pressure was more forecastable and scenario-based than the acute, human-facing crises confronting public-sector leaders, consistent with an appraisal of adversity as a relatively manageable operational challenge rather than an acute, human-facing threat. This organizational and psychosocial complexity in adversity exposure and capacity for resilience learning represents an important avenue for future research.

Finally, the Markov chain modelling’s projections, interpreted cautiously given the missing-data exclusions described above (see Results and Limitations), suggest a comparatively slow projected rate of progression toward behavioral change and resilience development across the full resilience sprints, with the long-run stationary distributions remaining concentrated at the reaction and learning levels even under indefinite continuation of the training. However, evaluating learning outcomes in leadership programs is inherently complex, as both participants and observers often struggle to determine whether, what, and with what awareness leaders learn (Day et al., 2021). Leaders may also reflect unconsciously and fail to recognize their behaviors as resilience, even though learning, for instance by adopting new perspectives and valuing others’ opinions fosters innovative problem-solving and strengthen resilience (Förster et al., 2022a). Future resilience studies should continue to explore how such dynamic mechanisms can be measured reliably and efficiently, ensuring that evaluations reflect both the complexity of short-term and long-term resilience processes and the realities of leaders’ work environments.

### Context: intervention facilitators and barriers

The findings indicate that contextual conditions across organizational, social, and individual levels shaped leaders’ engagement and learning processes during the intervention.

At the organizational level, high trainer commitment and a psychologically safe HR/leader community functioned as important facilitators. This aligns with research emphasizing that contextual conditions and experiential learning environments are essential for developing resilience capabilities (Duchek, 2020; Kolb and Kolb, 1984; Lengnick-Hall et al., 2011). Being gathered and repeatedly exposed to structured adversity-based exercises supported sense-making, responsiveness, and the development of shared mental models, mechanisms central to organizational resilience. Training resilience strategically builds organizational endowments (Williams et al., 2017), strengthens interpersonal connections through shared knowledge and interaction patterns (Lengnick-Hall et al., 2011) and maintaining the health of leaders in times of crisis (Förster and Duchek, 2022). Training practices are now recommended in crisis management and leader-oriented resilience training, where exposure to uncertainty strengthen adaptive capacity and prevents leaders from falling into panic or neglect of future crises (Förster and Duchek, 2022; James and Wooten, 2022). Leaders practiced situational recalls and “what-if” scenarios with expected uncertainty to balance experience in micro-strategic conversations (Van der Heijden and Heijden, 2005), thereby activating resilience potential in both structured and self-directed formats. The intervention’s design, which combined collective sessions with individual sprints, provided a full “learning-by-doing” cycle and learning space (Kolb and Kolb, 2005). However, the persistent tension between what could be called “training to work” and “working to train”, reveals an experiential learning paradox: leaders were offered to practice resilience in real time while simultaneously upholding operational responsibilities. These dual demands are not merely contradictory but mutually constitutive, and when alignment between learning and work falters, the potential of strategically “experiential resilience learning” becomes constrained, particularly under crisis conditions where cognitive bandwidth and organizational stability are already under pressure. Learning seen as a multilevel system (Sitzmann and Weinhardt, 2018) must take into account what happens before, during, and after training (Salas et al., 2012). The Markov modelling predicted sustained positive reactions and learning in sprints—which should be interpreted cautiously given the missing-data exclusions—but further indicated slower progression toward behavioral change, underscoring the importance of optimizing the overall learning context for long-term supported resilience development. Making the training mandatory alongside other core leadership development demands could perhaps strengthen engagement, as seen in mandatory resilience training across blue-light and military organizations, such as Comprehensive Soldier Fitness (Lester et al., 2011), however, unlike these high-reliability, command-structured contexts, mandating training for civilian leaders could perhaps undermine autonomous motivation (Ryan and Deci, 2017), deepen the “training to work” versus “working to train” tension identified above, and add further demands during already-compounding organizational pressure in times of crisis. Nearly half of the leaders struggled to participate without disrupting their work, and this challenge increased over time, potentially leading to inadequacy and guilt in both contexts. This reflects broader evidence that those who would benefit most from resilience training are often the least able to engage consistently due to workload and stress (Cheshire et al., 2017; Cleary et al., 2018). The dynamic shift in perceived timing appropriateness, from half of participants finding the timing suitable to only one third, with 43% moving to a neutral position, illustrates how availability and preferences fluctuate over time. Further it highlights the complexity and difficulty of aligning HRM practices with volatile leadership schedules in private and public organizations. Even flexible scheduling options and adjustments proved insufficient, exposing a gap between planned HRM practices and the nonlinear realities of leading and learning from crisis contexts. These findings reinforce calls for adaptive and context-sensitive HRM approaches to resilience (Förster and Duchek, 2022; Williams et al., 2017).

At the social level, the intervention activated collective interpretation processes that supported leaders’ ability to manage uncertainty. The consistently high experience of a free and open atmosphere, in a systematically organized leader forum with opportunities to share challenges and strategies, facilitated psychological safety, which is an established enabler of resilience (Edmondson, 1999; Vuori, 2023). Such shared sense-making processes help employees interpret unexpected events and expand resilience capabilities (Weick, 1995). Leaders reported that learning something new during the structured sprints and exchanging experiences with peers were particularly valuable, echoing calls for HRM practices that integrate cognitive, behavioral, and contextual elements in resilience development (Lengnick-Hall et al., 2011). Organizational learning practices that encourage shared interpretations of environmental signals, strategies for dealing with challenges, and provide opportunities to voice concerns of how it is to be a leader in turbulent times, can strengthen leaders’ sense of not being alone (Vuori, 2023). Collegial acceptance outside the formal sessions further strengthened this social context, suggesting that horizontal peer resources beyond the training community can function as additional resilience enablers. At the social level, trainer engagement also played a key role. External trainers onboarding and supporting HR-trainers were perceived as highly engaged, and internal trainer commitment increased substantially over time. Sustained support from competent trainers not only meets high performers’ basic psychological needs for self-motivation but also strengthens their resilience (Trigueros et al., 2019). This highlights the importance of HRM resources in instructor-led resilience training.

At the individual level, motivation emerged as a critical yet vulnerable factor in resilience processes. Although initial motivation was high, it declined substantially over time, mirroring patterns observed in longer interventions (Sitzmann et al., 2009). From a Self-Determination Theory perspective (Gagne and Gagné, 2014; Ryan and Deci, 2017), voluntary participation and peer-based experiential learning supported autonomous motivation, but competing demands, perceived pressure to train, or feelings of incompetence with a new topic could perhaps have undermined intrinsic motivation as controlled motivation entails pressure to think, feel, and act in prescribed ways (Ryan and Deci, 2017) which could be experienced when being educated and trained. Evidence similarly shows that motivation can be decisive for achieving positive psychological outcomes, as demonstrated in military resilience training where participant motivation played a key role in mental health improvements (Niederhauser et al., 2022). These insights help explain why different resilience interventions show limited or diminishing effects on wellbeing, psychosocial adjustment, and performance (Robertson et al., 2015; Scheuch et al., 2021; Vanhove et al., 2016) and why employees may revert to pre-training levels when motivation declines (Sarkar and Fletcher, 2017). In this study, the observed drop in leaders’ motivation calls for supportive, continued engagement and long-term transfer of resilience practices. However, motivation remains an underexplored yet central predictor of outcomes in resilience training research (Vanhove et al., 2016). Across more conventional leadership development studies, motivation is rarely examined empirically, despite evidence linking higher motivation to stronger transfer intentions and training effectiveness (Blume et al., 2010; Lauzier and Mercier, 2018; Ma and Chang, 2013). For future empirical leader resilience studies motivation is therefore crucial to explore further. In sum, the contextual results emphasize that leaders’ perceived resilience development depends on the dynamic interplay between organizational conditions, social support structures, and individual motivation for training.

Taken together, the mechanism-level patterns are unlikely to be independent of implementation and context: the sprint-format concerns raised in Implementation above (particularly around number and duration) plausibly constrained the sustained, repeated engagement that behavior- and results-level change requires, while the declining motivation reported above may in turn reflect leaders’ own progressive sense, across the program, that reaction and learning were not translating into visible behavior change—consistent with the interdependence between components anticipated in Methods. Resilience initiatives must therefore be designed in multi-level perspectives that not only provide experiential learning resources but also ensure the contextual training stability and motivational support required for leaders to engage, apply, and sustain resilience practices over time addressing how the organization needs to work, adapt, and survive at managerial levels throughout future crises.

### Implications for research and practice

As a process evaluation of a relatively small, non-randomized sample without a validated resilience outcome measure (see Limitations), the following implications are offered as evidence-informed practice considerations rather than established, generalizable recommendations.

A substantial gap remains in the organizational resilience training research, particularly for managerial levels, despite its growing urgency in times of crisis (Scheuch et al., 2021). Given the complexity of resilience processes, evaluations should incorporate contextual moderators and real-world dynamics rather than rely solely on single-variable RCT designs, which risk overlooking essential mechanism by treating them as confounding factors to be controlled for. This study illustrates that leaders’ self-reported psychosocial resilience consists of a spectrum of adaptive responsive processes emerging through leaders’ interactions with diverse adverse situations. Such intervention complexity requires identifying and reporting contextual factors that, although indirect, critically may shape implementation and act as mechanisms of change (Damschroder et al., 2009; Moore et al., 2015). Real-world evaluations can strengthen both relevance and generalizability (Coles et al., 2020), as illustrated here across polycrisis conditions in two organizations, though the small number of organizations and participants limits how far these specific findings generalize beyond this context. Moreover, agile approaches, such as developing contextual questionnaires (Moore et al., 2015), may help capture dynamic resilience factors in pressured environments as demonstrated in this study, where several themes emerged during the crisis-affected intervention. Our findings highlight that attention to initial program theory, combined with sensitivity to local contextual shifts, is essential for design flexibility, face validity, and more complete knowledge building about resilience processes and outcomes. Future long-term HRM resilience studies should therefore evaluate dynamic learning and development mechanisms and apply innovative multi-method designs to identify contextual facilitators (to strengthen programs) and barriers (to improve programs) that shape leader resilience.

Seven resilience-specific considerations follow from these findings for HR partners and leaders designing and engaging with similar programs. First, because experiential resilience training (adapted from Kolb’s cycle) draws directly on the leaders’ lived adversity, polycrisis conditions introduce structural volatility into the training content itself, not only its scheduling: programs should build in adaptive slack in sprint content and cadence, not only timing, to absorb unpredictable shifts in adversity. Second, resilience training could risk individualizing what is, in practice, a shared organizational vulnerability (Cabanas and Illouz, 2019; Chandler and Reid, 2016). However, responsibility is better understood as a negotiated, both-and undertaking (Kuntz et al., 2017; Ungar and Jefferies, 2021), with HR securing protected time and reduced concurrent demand while leaders engage in the reflective work training cannot do on their behalf. Third, the shared, psychologically safe gatherings in which leaders exchanged experiences of adversity, identified here as a key facilitator, should be treated as a resilience-building resource in its own right and preserved even where other elements must be adapted. Fourth, motivation for resilience training may behave differently than motivation in conventional skills training: the cognitive and emotional narrowing accompanying stressful polycrisis conditions may simultaneously undermine leaders’ capacity to engage with that same training, producing a paradox in which motivation is most needed and least available exactly when adversity peaks. Concrete solutions include lowering sprints’ reflective demands during crisis peaks, leaning more heavily on the peer forum when reflective capacity is depleted, and reframing training at peak pressure as an immediate coping resource rather than a competing development obligation. Fifth, the slow projected progression from reaction and learning toward behavior change (see Mechanisms of impact) suggests HR partners should set realistic expectations for when behavioral change becomes visible, treating early evaluations as indicators of engagement rather than evidence of limited effectiveness, and planning program duration accordingly. Sixth, the self-assessment difficulty identified above (e.g., the participant quote) suggests that resilience-specific psychoeducation, however brief, before the first sprint may meaningfully strengthen the validity of subsequent self-report evaluations. Without an explicit, shared understanding of what resilience for leaders concretely means and what each Kirkpatrick level asks participants to evaluate, Level 3–4 self-ratings risk reflecting diverse construct understanding as much as variable resilience development itself. Front-loading a short definition and knowledge on the measurement component, rather than beginning directly with introducing the experiential exercises, could therefore serve both a pedagogical and a methodological function in future programs. Seventh, the marked contrast between how leaders evaluated the resilience sprints´ internal structure (49% agreement) versus their number (20% agreement) suggests that the core value of the format lies in its content composition rather than its frequency. Future programs facing similar chaotic circumstances and ongoing time pressure could therefore reduce sprint frequency without necessarily sacrificing the elements leaders found most valuable, offering HR partners a concrete lever for reducing the training-to-work/working-to-train tension without redesigning the format itself.

Beyond these practice-level considerations, future research should further disentangle resilience process from resilience outcome measurement (Britt et al., 2016) and examine how training format, together with emerging contextual factors, causally affects resilience training processes and motivation to develop resilience in long-duration psychosocial resilience programs for crisis-affected leaders.

### Limitations and strengths

This HRM resilience training study has several limitations. First, it included leaders from different managerial levels but only from one private-sector and one public-sector organization within the same country, limiting contextual diversity. Recruitment during an intense crisis period further narrowed access to organizations and participant representation. In addition, the final analytic samples (35–42 participants, depending on component, down from an initial 48) were small relative to the number of subgroup comparisons reported by organization and resilience category. We could have avoided this limitation by reporting only pooled, sample-wide results, however, we judged that showing between-organization and between-category variation was essential to capture the contextual differences central to this study’s process evaluation aim, even though it comes at the cost of reduced statistical power and generalizability for these more granular findings. Second, the focus on three primary resilience factors may restrict transferability to programs targeting other resilience dimensions. Third, the analysis relied primarily on survey data and secondarily on log entries. Incorporating interviews with participants, HR trainers, superiors, and employees, as well as examining communication between HR trainers and external consultants, could have strengthened internal validity. Fourth, the tailored questionnaire developed specifically for this study, grounded in observations and discussions, was necessary and ethically appropriate under turbulent conditions but may have influenced process evaluation outcomes and limited comparability with established instruments. Both the implementation and contextual questionnaires were face-validated through the early observation and discussion process described above but were not subjected to formal psychometric validation. Fifth, Kirkpatrick Level 4 (“Results”) was operationalized through leaders’ self-report of resilience development supported by the training, rather than through independently observed behavior or a validated resilience outcome measure. While this level appropriately targets the training’s own stated goal of resilience development rather than organizational performance, its evidentiary strength is therefore comparable to the self-report items at Levels 1–2 rather than an independently validated result. Despite these constraints, the multi-methods design and data triangulation provided a explore basis for examining resilience-building processes in complex interventions. Overall, these limitations reflect the realities of conducting research during crisis conditions, while the methodological choices enabled a comprehensive understanding of leaders’ experiences of resilience training and contributed new insights across several research fields.

## Conclusion

This study offers process-level evidence for the strategic HRM importance of resilience training for leaders operating amid constant change and emerging crises. Organizations must not only learn from adverse events but also translate these lessons into leader values, behaviors, and practices (Duchek, 2020). Viewing resilience training as a dynamic learning process highlights how such training aims to help leaders develop adaptive capabilities that enable constructive functioning under extreme conditions. The findings illuminate the complex mechanisms shaping leaders’ engagement with resilience training and emphasize the need for proactive design and implementation in leader-focused interventions. Strategic HRM resilience training, supported by HR business partners and consultants, plays a crucial role in engaging leaders with training that targets cognitive, emotional, behavioral, and social resilience capabilities. Evaluating design, implementation, and contextual factors provides multilevel insights that contribute to crisis leadership, resilience, and management research. Although long-term engagement in resilience programs may challenge motivation and prioritization, such programs offer leaders valuable opportunities to reflect and make sense of adversity in safe HR-facilitated spaces while facing it.

Humans possess a remarkable capacity to learn from stressful circumstances. As organizations face increasing uncertainty, nurturing resilience at managerial levels becomes essential. Investing in leader wellbeing and professional development strengthens strategic human capacity, enhances adaptability, and supports navigation of complex and adverse conditions, ultimately securing a competitive advantage in an ever-changing global landscape. These insights provide sustainable organizational futures grounded in the human core that drives them—simultaneously vulnerable and profoundly strong as they are. These insights provide a foundation for further exploration of leader resilience training and support HRM scholars and practitioners seeking to help organizations and their leaders engage realistically with crises rather than retreat from them.

## Data Availability

The original contributions presented in the study are included in the article/Supplementary material, further inquiries can be directed to the corresponding author/s.
