# Supplementary material for: Preparing to lead in polycrisis: a multi-method process evaluation of HR-driven psychosocial resilience training for leaders
Source: Front Psychol. 2026 Oct 23;17:1900181. doi: 10.3389/fpsyg.2026.1900181 (PMC13739556; doi:10.3389/fpsyg.2026.1900181)
Supplement: Supplementary file 1 [file Data_Sheet_1.pdf]

Appendix 1: Mixed method matrix

	Type of instrument	Purpose	Type of evaluation	Data collection and sample	Instrument and analysis method	Framework element
1.	Participant manual score sheet/SMS survey	Training evaluation of implemented sessions for evaluating effective training and training effectiveness	Summative/ formative	Collected continuous from attended participants, session 1-20. Scored on posters during sessions or SMS. Not fully anonymous	Quantitative analysis of Kirkpatrick Scale by analyst	Mechanism
2.	Participant manual score sheet/SMS survey	Implicit evaluation of 3 overarching resilience promoting factors implemented through curriculum	Summative/ formative	Collected continuous from attended participants, session 1-20. Anonymous	Quantitative analysis of Kirkpatrick Scale by analyst	Mechanism
3.	Participant survey	Assessment of motivation, engagement, and contextual emerging factors	Summative/ formative	Collected from all participants, repeated (T2-T3). Anonymous	Self-developed questionnaire. Descriptive statistics performed on all items by analyst	Context, implementation, mechanism
4.	Participant survey	Assessment of deliverance format fit to a turbulent leader context after the training implementation	Formative	Collected from all participants (T3). Anonymous	Self-developed questionnaire. Descriptive statistics performed on all items by analyst	Implementation
5.	HR evaluation and comment sheet	Primary function as a dialogue tool on the resilience trainer education for possible adjustments (piloting) within the scope of research design: Check of program logic consistency and assessment of continuous HR trainer satisfaction with program content and contextual validation and relevance for local leader contexts	Summative	Collected from all HR resilience trainers on the resilience trainer education (or by email afterwards). Non-anonymous	Self-developed questionnaire. Reviewed by main researcher for small adjustments before the training period with leaders and for informative process evaluation purposes	Implementation
6.	Facilitator attendance sheets of weekly sessions	Assessment of participation (dose of curriculum/exposure to knowledge and skill enhancing sessions) and reach of program	Summative/ formative	Collected weekly from facilitators on sites in the training period. Non-anonymous	Scores of attendances for each session were entered into an Excel sheet and calculated by main researcher for blinded analysis by analyst (demographics used in RCT study)	Implementation

7.	Facilitator/ research log	To assess the delivery of organizational resources: HR fidelity of curriculum implementation, quantity of deliverance sessions/maintenance of leader network, and emerging contextual adaptions. Monitoring/program management of the local organizational projects including HR issues for ongoing sparring	Summative	Collected during the training period (facilitator observations on sites, reflections, relevant HR conversations and correspondence). Blinded to HR and participants	The qualitative data were reflected and reviewed by main researcher for process evaluation informative/additive purposes during and after the intervention	Context, implementation, mechanism
