# Supplementary material for: Preparing to lead in polycrisis: a multi-method process evaluation of HR-driven psychosocial resilience training for leaders
Source: Front Psychol. 2026 Oct 23;17:1900181. doi: 10.3389/fpsyg.2026.1900181 (PMC13739556; doi:10.3389/fpsyg.2026.1900181)
Supplement: Supplementary file 2 [file Data_Sheet_2.pdf]

Appendix 2. Questionnaires used in the process evaluation

Component 1: The resilience training implementation

Type of instrument	Items	Response scale	Research focus
Participant survey (contextualized)	"The number of sprints in the resilience course (20 sprints) has seemed appropriate to me"	5-point Likert scale	Program format
	"The interval between sprints (approx. one week) in the resilience course has been appropriate for me"		
	"The duration of a sprint (60 minutes) has been appropriate for me to spend on resilience training"		
	"The distribution of the sprint elements (joint review of the past week, new knowledge, time with my training book and dialogue with others) has been appropriately distributed over the 60 minutes"		

Note. Items were completed at T3 on the 5-point Likert scale (Strongly disagree – Strongly agree).

Component 2: The resilience training mechanism of impact (Kirkpatrick)

Type of instrument	Items	Response scale	Research focus
Participant manual score sheet / SMS survey (Kirkpatrick Model, contextualized)	"How far have I come?"<br>1. It was a good sprint last time.<br>2. I learned something last time.<br>3. I have tried something new since last time.<br>4. The training has supported resilience development.<br><br>Other comments	Single-choice item + optional open-ended response	Learning and development (effective training and training effectiveness)

Note. Items were completed at the end of each facilitated/self-directed resilience sprint.

Component 3: Contextual factors

Type of instrument	Items	Response scale	Research focus
Participant survey (contextualized)	"It has worked well for me that we have been gathered for the resilience training".<br>"The time of day we have met has suited me well".<br>"I have been able to prioritize resilience training without significantly disrupting my work".<br>"I have experienced acceptance from my colleagues when I have had to leave for resilience sprints".<br>"I have experienced a free and open atmosphere when we have shared knowledge and experiences about resilience".<br>"I have been motivated to develop my resilience during the course".<br>"The external trainer has seemed engaged in the training".<br>"The internal trainers have been committed to the training".	5-point Likert scale	Contextual emerging factors

Note. Items were completed at T2 and T3 on the 5-point Likert scale (Strongly disagree – Strongly agree).
