# Supplementary material for: Preparing to lead in polycrisis: a multi-method process evaluation of HR-driven psychosocial resilience training for leaders
Source: Front Psychol. 2026 Oct 23;17:1900181. doi: 10.3389/fpsyg.2026.1900181 (PMC13739556; doi:10.3389/fpsyg.2026.1900181)
Supplement: Supplementary file 3 [file Data_Sheet_3.pdf]

Appendix 3. Transition Matrices for Contextual Factors (Items 1C-8C)

This appendix presents the transition matrices for all eight contextual factors. Each matrix illustrates how participants' responses changed between T2 (rows) and T3 (columns). The row percentages indicate the distribution of T3 responses within each T2 category, while the column percentages show the proportion of participants ending in each T3 category. The response categories are coded as −1 = Disagree, 0 = Neutral, and 1 = Agree, corresponding to the categories used in Table 4 in the main text.

Item 1-C. "It has worked well for me that we have been gathered for the resilience…"

T2 → T3	−1	0	1	Row %
**−1**	3	1	1	14%
**0**	2	5	1	23%
**1**	0	2	20	63%
**Column %**	14%	23%	63%	**N = 35**

Item 2-C. "The time of day we have met has suited me well."

T2 → T3	−1	0	1	Row %
**−1**	3	7	1	31%
**0**	1	3	2	17%
**1**	4	5	9	51%
**Column %**	23%	43%	34%	**N = 35**

Item 3-C. "I have been able to prioritize resilience training without significantly disrupting my work."

T2 → T3	−1	0	1	Row %
**−1**	13	2	1	46%
**0**	4	2	2	23%
**1**	3	0	8	31%
**Column %**	57%	11%	31%	**N = 35**

Item 4-C. "I have experienced acceptance from my colleagues when I have had to leave for resilience sprints."

T2 → T3	−1	0	1	Row %
**−1**	0	1	1	6%
**0**	0	9	3	34%
**1**	0	5	16	60%
**Column %**	0%	43%	57%	**N = 35**

Item 5-C. "I have experienced a free and open atmosphere when we have shared knowledge and experiences about resilience."

T2 → T3	−1	0	1	Row %
**−1**	2	0	0	6%
**0**	0	4	3	20%
**1**	0	2	24	74%
**Column %**	6%	17%	77%	**N = 35**

Item 6-C. "I have been motivated to develop my resilience during the course."

T2 → T3	−1	0	1	Row %
**−1**	2	2	0	11%
**0**	1	6	3	29%
**1**	2	8	11	60%
**Column %**	14%	46%	40%	**N = 35**

Item 7-C. “The external trainer has seemed engaged in the training.”

No transition matrix was calculated, as the external trainer only supported the initial phase.

Response	Count	%
**−1**	3	7%
**0**	3	7%
**1**	35	85%
**Total**	41	—

Item 8-C. “The internal trainers have been committed to the training.”

T2 → T3	−1	0	1	Row %
**−1**	0	1	2	9%
**0**	2	3	6	31%
**1**	0	2	19	60%
**Column %**	6%	17%	77%	**N = 35**
